# Dual gene therapy with extracellular superoxide dismutase and catalase attenuates experimental optic neuritis

**Published:** 2007-01-05

**Authors:** Xiaoping Qi, William W. Hauswirth, John Guy

**Affiliations:** 1 Department of Ophthalmology, Molecular Genetics, University of Florida, College of Medicine, Gainesville, FL; 2 Department of Microbiology, University of Florida, College of Medicine, Gainesville, FL; 3 Neurology, University of Florida, College of Medicine, Gainesville, FL

## Abstract

**Purpose:**

To ameliorate experimental optic neuritis by combining scavenging of superoxide by germ line increases in the extracellular superoxide dismutase (ECSOD) and hydrogen peroxide by viral-mediated gene transfer of the human catalase gene.

**Methods:**

The human catalase gene inserted into recombinant adeno-associated virus (rAAV) was injected into the right eyes of transgenic mice overexpressing human ECSOD and wild-type littermates. Animals were simultaneously sensitized for experimental autoimmune encephalomyelitis (EAE) and then sacrificed one month later. The effects of antioxidant genes (ECSOD and catalase) on the histologic lesions of EAE were measured by computerized analysis of myelin area, optic disc area, extent of the cellular infiltrate, cerium derived H_2_O_2_ reaction product and extravasation of serum albumin detected by immunogold.

**Results:**

Combined scavenging of H_2_O_2_ and superoxide with ECSOD and catalase suppressed demyelination by 72%, 54% due to catalase, and 19% due to ECSOD. Disruption of the blood-brain barrier was reduced 63% by the combined effects of catalase and ECSOD, 35% due to catalase and 29% due to ECSOD.

**Conclusions:**

Transgene modulation of antioxidant enzyme defenses against both superoxide and its metabolite H_2_O_2_ provide a substantial suppressive effect against EAE in the optic nerve that may be a new therapeutic strategy for suppression of optic neuritis and multiple sclerosis.

## Introduction

Experimental autoimmune encephalomyelitis (EAE) is an autoimmune inflammatory disorder leading to primary central nervous system demyelination. EAE has been frequently used as an animal model for testing treatments against multiple sclerosis (MS) [1-16]. The optic nerve is a frequent site of involvement in both EAE and MS [17-23]. Reactive oxygen species (ROS) such as superoxide, hydrogen peroxide, nitric oxide and peroxynitrite are mediators of demyelination and disruption of the blood-brain barrier (BBB) in EAE [24-31]. The role ROS play in altering BBB permeability and demyelination has been inferred from the beneficial effect of monotherapy with free radical scavengers or antioxidants on EAE [27-31]. ROS scavengers include catalase and superoxide dismutase (SOD). SOD dismutes superoxide to hydrogen peroxide (H_2_O_2_) and catalase detoxifies the H_2_O_2_ to H_2_O and O_2_.

In a prior study, we targeted a single ROS, hydrogen peroxide, for detoxification by catalase gene inoculationn [23]. It reduced demyelination of the optic nerve by 38%. An approximately one-third suppressive effect on disease activity is achieved by currently available treatments for MS by utilizing a single drug [32]. Some studies have suggested that combination therapy may have a better suppressive effect on MS than monotherapy [33,34] although this is not always the case [35]. Here, we attempt to further ameliorate EAE by assessing the additional protective effects on experimental optic neuritis of combining in vivo scavenging of superoxide by germ line increases in the extracellular superoxide dismutase (ECSOD) and scavenging of hydrogen peroxide by viral mediated gene transfer of the human catalase gene.

## Methods

### Recombinant adeno-associated virus

The adeno-associated virus (AAV) vector pTR-UF was used to accept the human catalase cDNA at the Not1 and Sal1 sites. The resulting pTR-CAT plasmid were amplified, then purified and packaged into serotype 2 rAAV. Briefly, recombinant AAV was purified through iodixanol step gradients and heparin-agarose affinity columns and assayed as previously described [36]. Each virus preparation contained 10^11^ to 10^12^ particles per milliliter and 10^9^ to 10^10^ infectious center units per milliliter.

### Induction of EAE and intraocular injections

Two μl of recombinant adeno-associated virus (rAAV) catalase were injected into the vitreous cavity of 20 transgenic ECSOD mice, overexpressing human extracellular superoxide dismutase (ECSOD; a generous gift of Dr. James Crapo) and 20 wild-type littermates were also injected with AAV-catalase as controls. Briefly, ECSOD transgenic mice were constructed by injection of DNA containing the entire human ECSOD cDNA driven by a human β-actin promoter that was injected into pronuclei of fertilized eggs that were isolated from mice [(C57BL/6xC3H)F1x(C57BL/6xC3H)F1]. Surviving eggs were implanted into pseudopregnant foster mothers to generate offspring containing the ECSOD transgene. Mice expressing human ECSOD were identified using Southern blot analysis of DNA extracted from the tail and probed with the entire human ECSOD cDNA [37,38]. EAE was induced in the mice by sensitization with 0.2 cc of ultrasonically homogenized spinal cord emulsion in complete Freunds adjuvant (Difco, Detroit, MI) that was injected subdermally into the nuchal area [30]. Mice were maintained in veterinarian-supervised animal care facilities that are fully accredited by the American Association of Laboratory Animal Science and they were humanely cared for in full compliance with ARVO guidelines.

### Immunobloting and immunohistochemistry

Retinal ganglion cells (RGC-5) were grown in Dulbecco's Modified Eagle Medium (DMEM; Fisher Scientific) supplemented with 10% heat-inactivated fetal bovine serum and 1% penicillin streptomycin (Sigma) at 37 °C with 5% CO_2_. Cells were grown in 15 cm dishes that were infected with AAV containing the human catalase cDNA at multiplicities of infection (MOI) of 5,000 particles per cell. Controls were infected with AAV-GFP. Two days after AAV infections, cells were harvested. Briefly this involved washing the trypsinized cells in cold PBS, then manually homogenizing them. For immunodetection, 15 mg of homogenated protein was separated on a 10% SDS polyacrylamide gel and electro-transferred to a polyvinylidene fluoride membrane (BioRad, Hercules, CA). We immunostained the membrane with monoclonal anti-catalase antibodies (Sigma-Aldrich, St. Louis, MO, C0979, mol wt, 60 kDa) and then goat anti-mouse IgG horseradish peroxidase (HRP) conjugated secondary antibodies (Sigma-Aldrich). We detected complexes using the enhanced chemiluminesence (ECL) system (Amersham Pharmacia Biotech, Piscataway, NJ). Anti-mouse β-actin antibody was used as an internal control for protein loading.

One month after AAV and EAE inoculations, mice were overdosed with sodium pentobarbital (0.3 mg/g body weight). They were then perfused by cardiac puncture with fixative consisting of 4% paraformaldehyde in 0.1 M PBS buffer (pH 7.4). The eyes with attached optic nerves were dissected out of ten ECSOD mice and ten littermates. The specimens were further processed by immersion fixation in 2.5% gluteraldehyde, postfixed in 1% osmium tetroxide, 0.1 M sodium cacodylate-HCl buffer (pH 7.4), 7% sucrose in the cold, and then dehydrated through an ethanol series to propylene oxide, infiltrated, and embedded in epoxy resin that was polymerized at 60 °C overnight. For immunocytochemistry, tissue specimens from the other ten ECSOD mice and ten littermates were postfixed in 5.0% acrolein, 0.1 M sodium cacodylate-HCl buffer (pH 7.4) and 7% sucrose and then dehydrated through an ethanol series and embedded in LR-White (Ted Pella, Redding, PA) that was polymerized at 50°C overnight. Semi-thin longitudinal sections (0.5 μm) of the optic nerve head and retrobulbar nerve were stained with toluidine blue for light microscopic examination. Ultrathin sections (90 nm) were placed on nickel grids for immunocytochemistry. Nonspecific binding of antibodies was blocked by 5% normal goat serum in 0.01 M Tris-buffered saline, (pH 7.2) or 2% teleost gelatin and 2% nonfat dry milk in 0.01 M TBS (pH 7.2) with TBST for 30 min for albumin immunostaining. They were then reacted with a rabbit anti-albumin antibody or an ECSOD antibody (a generous gift of Dr. Stephan Marklund) that recognizes the human ECSOD [39], but not the murine ECSOD (personal communications with Dr. Marklund) in the same buffer for 2 h at room temperature.

After washes in 0.1 M PBS, the specimens were reacted with the secondary goat anti-rabbit IgG antibodies conjugated to 10 nm gold or Cy3 for immunofluorescence microscopy. After washes in buffer, grids were rinsed in deionized water. For examination at low magnification transmission electron microscopy, the immunogold particles were enlarged by silver enhancement using a kit (Ted Pella, Redding, PA) according to the manufacturer's specifications. To check for nonspecific binding of the secondary antibody, control specimens were incubated in the buffer, followed by the gold-labeled or Cy3 labeled antibody. Immunolabeled and control specimens were photographed by transmission electron microscopy without poststaining.

### Morphometric analysis

Morphometric analysis was performed in masked fashion as previously described [23]. Briefly, images of toluidine blue stained sections of the optic nerve were captured with a video camera mounted on a light microscope and then the digital image was entered into computer memory. After initial calibration with a stage micrometer, the optic nerve head areas were manually traced using NIH IMAGE software and a MacIntosh Computer (Apple, Cupertino, CA). The number of glial cells and inflammatory cells in the retrobulbar optic nerve were also quantitated by thresholding of the darker staining cell nuclei. Using electron microscopy, identification of glial cells in the optic nerve was based on morphologic criteria. Fibrous astrocytes were identified by their round or elliptical nuclei with few clumps of chromatin in a relatively light karyoplasm that was surrounded by a voluminous pale cytoplasm with long processes and glial filaments. Inflammatory cells were identified by the prominent clumping of nuclear chromatin, ribosome rich cytoplasm that was clearly more electron dense than that of astrocytes and the presence of phagosomes with engulfed myelin debris.

Optic nerve specimens were examined without poststaining using a Hitachi H-7000 transmission electron microscope (Tokyo, Japan) operating at 75 kV. Photographs were made at a magnification of 2,500X. For quantitative analysis, micrographs of each optic nerve were digitized into computer memory by using a UMAX scanner (UMAX Data Systems, Fremont, CA). Extravasated serum albumin immunogold or H_2_O_2_ derived cerium perhydroxide were quantitated by thresholding the respective elements. Mean particle counts for each nerve were obtained by taking the mean value of the 10 micrographs. Each mean value was expressed as the number of elements per unit area. The extent of demyelination was quantitated by threshold measurements of the electron dense myelin sheaths that were derived from the axonal transmission electron micrographs for each optic nerve. Again using NIH IMAGE software, we utilized the threshold feature to outline the myelin sheaths for each micrograph. Glial or inflammatory cells that were also highlighted in the thresholded micrographs were removed manually by using the eraser function. To calculate the outlined myelin area we used the software "analyze" feature. Increases in myelin sheath area (less demyelination) thereby indicated a beneficial treatment effect. Grouped student t-tests were used to assess differences in the myelin areas, optic nerve head areas, optic nerve cell counts, hydrogen peroxide reaction product and extravasated albumin immunogold between the transgenic ECSOD mice and the wild-type littermates.

## Results

### ECSOD and catalase expression

**Figure 1:**
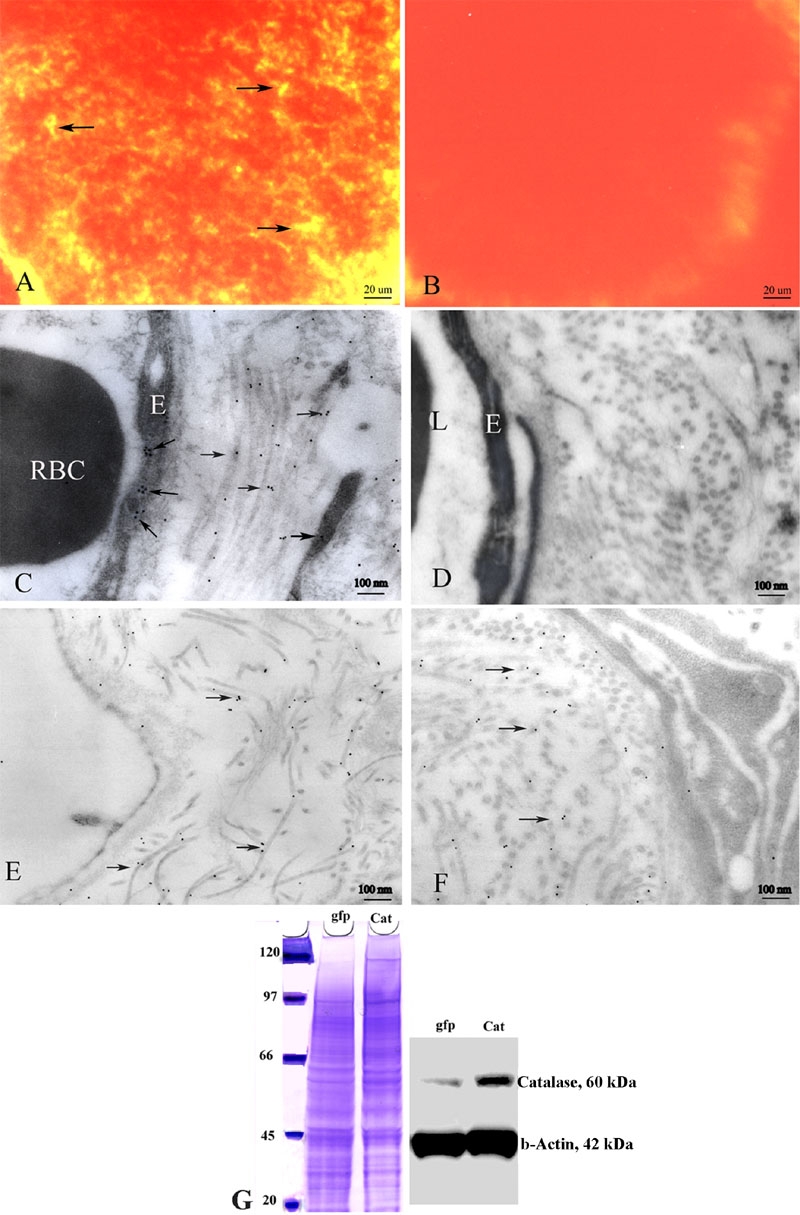
Expression of extracellular superoxide dismutase and catalase. Immunofluorescence micrographs show expression of the human ECSOD (arrows) in the optic nerve of a representative transgenic ECSOD mouse (**A**), but it is absent in the optic nerve of a wild-type littermate (**B**). Transmission electron micrograph of the retrobulbar optic nerve of a transgenic ECSOD mouse reveals ECSOD immunogold (arrows) in the perivascular space and endothelia of the optic nerve (**C**), peripapillary choroid (**E**) and optic nerve sheath (**F**). Human ECSOD is absent in wild-type littermates (**D**). Immunobloting shows increased catalase expression in cultured retinal ganglion cells infected with rAAV containing the gene for human catalase, relative to control RGC-5 cells infected with AAV-GFP (**G**). E represents endothelial cell. RBC represents red blood cell. L represents lumen, Cat represents catalase, gfp represents green fluorescent protein.

Expression of the human ECSOD was evident in the optic nerves of transgenic ECSOD mice (Figure 1A), but not in the wild-type littermates (Figure 1B). As implied by its name, ECSOD immunogold localized to the perivascular space and endothelial cells in the optic nerve (Figure 1C) and peripapillary choroid (Figure 1E). It was also represented in the meninges comprising the optic nerve sheath of ECSOD mice (Figure 1F). Human ECSOD immunogold was absent in the perivascular space of the littermates (Figure 1D). This distribution of ECSOD mirrored the presence of H_2_O_2_ in the EAE optic nerve. Immunobloting showed that retinal ganglion cells infected with rAAV containing the gene for human catalase had increased catalase expression relative to controls infected with AAV-GFP (Figure 1G).

### Demyelination

**Figure 2:**
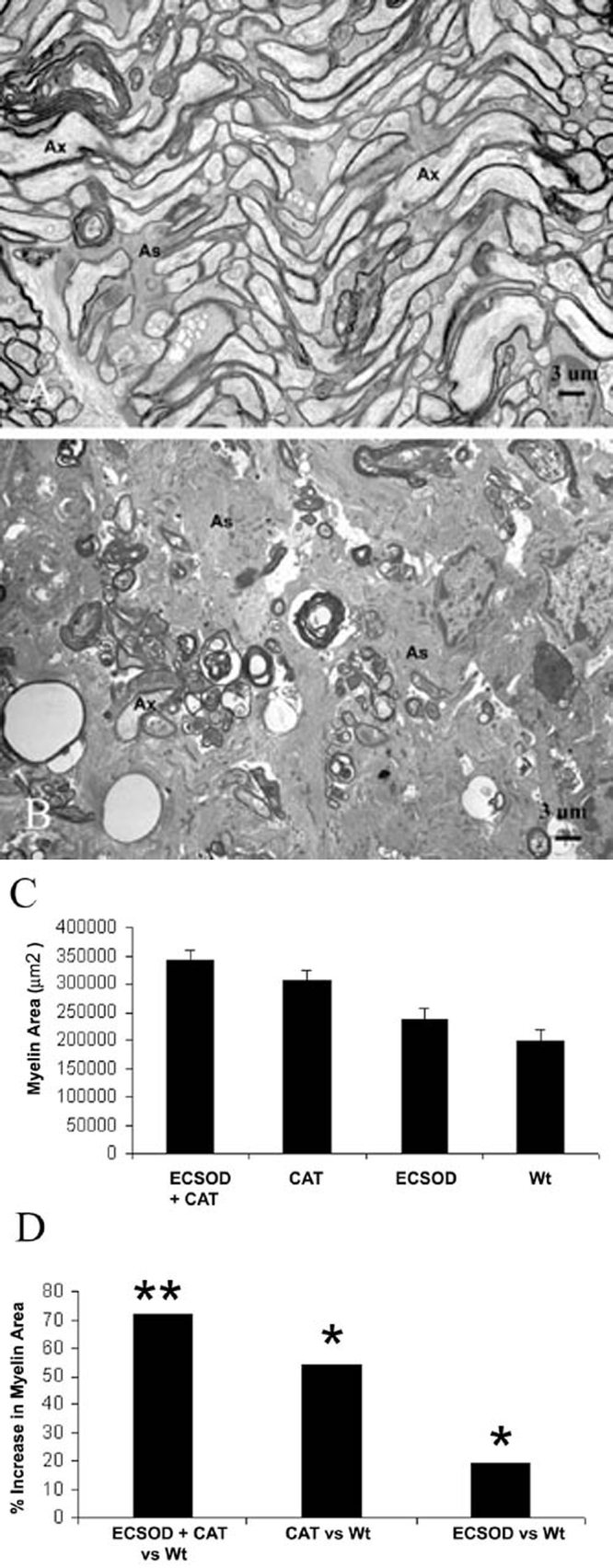
Suppression of demyelination. Representative transmission electron micrographs of the retrobulbar optic nerve show many normal fibers and substantially less demyelinated and thinly myelinated axons following rAAV-catalase inoculation of the right eyes of transgenic ECSOD mice (**A**), relative to the unprotected left eyes of wild-type littermates in whom marked fiber loss, naked axons and those with thin sheaths of myelin were prominent ultrastructural findings (**B**). The barplot shows mean myelin areas of the retrobulbar optic nerve protected by both ECSOD and catalase (ECSOD OD), catalase (Wt OD), ECSOD (ECSOD OS) and unprotected EAE (Wt OS; **C**). Barplot (**D**) illustrates the preservation of myelin induced by ECSOD and catalase, catalase or ECSOD relative to unprotected nerves. Asterisk (*) represents p<0.05, double asterisks (**) represents p<0.01, Ax represents axon, As represents astrocyte process.

Light microscopy of the EAE optic nerves revealed foci of demyelination, the hallmark of the histopathology of EAE and MS, was evidenced by loss of toluidine blue staining. This finding was seen to some degree in all animals sensitized for EAE. Transmission electron microscopy clearly demonstrated the benefits of anti-ROS gene therapy. Illustrative of the benefits of double protection, the right eyes of ECSOD mice that received the AAV-catalase gene inoculation had much less myelin fiber injury than the unprotected left eyes of wild-type mice. A representative micrograph of the right eyes of ECSOD mice further protected by catalase shows a near normal complement of optic nerve fibers with relatively preserved myelin lamellae (Figure 2A). These findings sharply contrasted with the unprotected left eyes of wild-type littermates where myelin and fiber loss was severe (Figure 2B). Here remaining axons were degenerating, enveloped by thin sheaths of myelin, or naked. Optic nerve fibers were replaced by a proliferation of astroglial processes.

Measurements of myelin area in wild-type mice not sensitized for EAE revealed a mean of 439,683 μm^2^. This value was comparable to the normal unsensitized ECSOD transgenic mouse with a value of 424,878 μm^2^. Relative to unsensitized wild-type mice, the myelin area of unprotected wild-type mice induced with EAE (Wt OS) was reduced by 55% (p<0.03). Quantitative analysis confirmed that in vivo scavenging of hydrogen peroxide by viral mediated catalase gene transfer and superoxide scavenging by ECSOD resulted in a mean myelin area of 340,236 μm^2^ (ECSOD OD), thus reducing demyelination by 72% relative to a value of 197,517 μm^2^ (Wt OS) for untreated wild-type mice with EAE (p<0.01; Figure 2C-D). This combined effect was greater than that of superoxide scavenging alone as ECSOD mice had 19% more myelin (less demyelination) with a mean myelin area of 235,123 μm^2^ (ECSOD OS) than the mean of 197,517 μm^2^ (Wt OS) for unprotected wild-type mice (p<0.05). It was also greater than the solo effect of catalase mediated scavenging of hydrogen peroxide with a mean myelin area of 304,190 μm^2^ (Wt OD) versus 197,517 μm^2^ (Wt OS) that reduced demyelination by 54% (p<0.01). Relative to unsensitized wild-type mice, myelin area was reduced by 23% even with combined scavenging (ECSOD OD). However, this difference was not statistically significant. Clearly, double protection against superoxide and H_2_O_2_ by ECSOD and catalase offered the best protection against the most desirable parameter sought after for treatment, amelioration of myelin fiber injury in the EAE optic nerve. The solo effect of catalase was better than that of ECSOD.

### Optic disc edema

**Figure 3:**
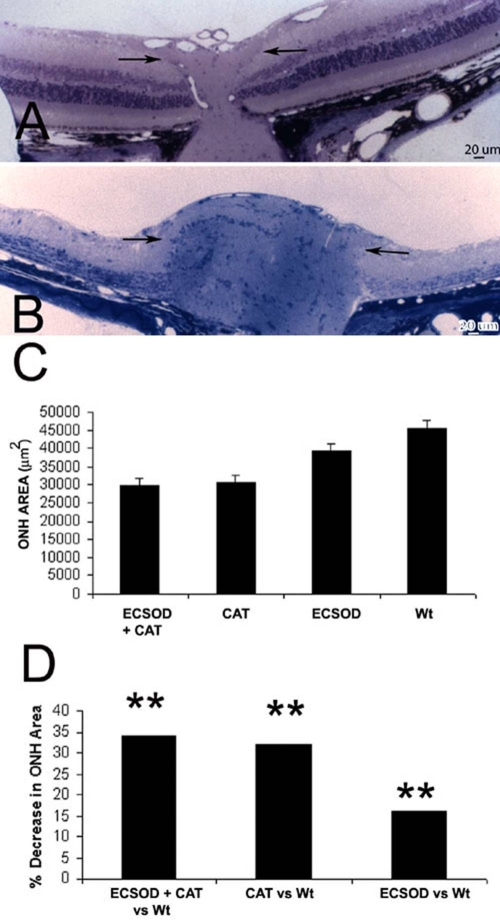
Suppression of optic disc swelling. Representative light micrographs showing catalase and SOD suppressed optic nerve head edema (**A**) relative to the unprotected optic nerve (arrows) exhibiting marked swelling of the optic nerve head (**B**). The barplot of mean optic nerve head (ONH) areas shows that optic nerve head swelling (smaller ONH area) was reduced by combined ECSOD and catalase (ECSOD OD) treatment, catalase treatment (Wt OD), ECSOD treatment (ECSOD OS), but it was greatest with no treatment (Wt OS; **C**). Barplot (**D**) illustrates the reduction in ONH swelling induced by ECSOD and catalase, catalase or ECSOD relative to unprotected nerves. Asterisk (*) represents p<0.05, double asterisks (**) represents p<0.01.

Optic disc edema, seen ophthalmoscopically in approximately one-third of patients with acute optic neuritis or multiple sclerosis, was evident in EAE animals in which lateral displacement of the peripapillary retina and filling of the optic cup indicated optic nerve head swelling at the light microscopic level. Relative to ECSOD and catalase-protected nerves (ECSOD OD; Figure 3A), optic nerve head swelling was most severe in the unprotected nerves of wild-type mice (Wt OS; Figure 3B). The combined effects of in vivo scavenging of hydrogen peroxide by viral mediated catalase gene transfer and superoxide scavenging by germ line increases of ECSOD reduced optic nerve head edema by 34% with a mean optic nerve head area of 29,821 μm^2^ (ECSOD OD) relative to wild-type mice with a mean value of 45,354 μm^2^ (Wt OS; p<0.01; Figure 3C-D). This combined effect was greater than that of superoxide scavenging alone. ECSOD mice had 16% less optic disc edema with a mean optic nerve head area of 38,092 μm^2^ (ECSOD OS) relative to a mean of 45,354 μm^2^ (Wt OS) for wild-type mice (p<0.05). Clearly, the additional benefit of ECSOD was not much greater than the solo effect of catalase that reduced optic disc edema by 32% with a mean optic nerve head area of 30,754 μm^2^ (Wt OD) relative to 45,354 μm^2^ for untreated eyes (Wt OS; p<0.01).

### Optic nerve cell count

**Figure 4:**
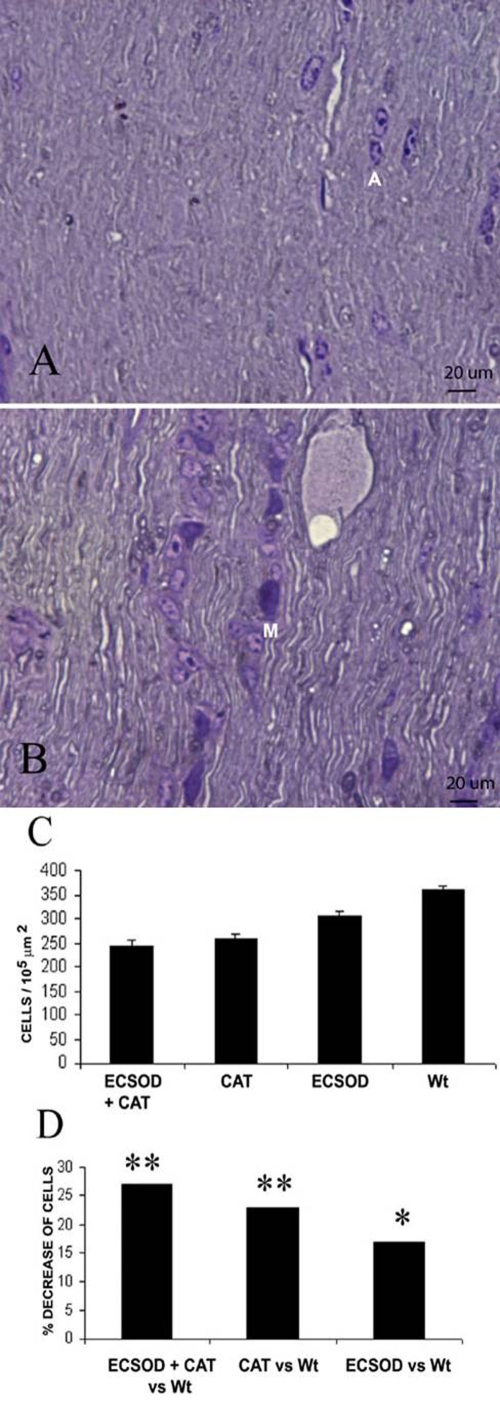
Suppression of cellular infiltration. Representative light micrographs show that cellular infiltration in the retrobulbar optic nerve is reduced by double protection with ECSOD and catalase (**A**) relative to the unprotected optic nerve (**B**). The barplot shows the mean optic nerve cell count of the retrobulbar optic nerve protected by both ECSOD and catalase (ECSOD OD), catalase (Wt OD), ECSOD (ECSOD OS) and unprotected EAE (Wt OS; **C**). Barplot (**D**) illustrates the reduction in the optic nerve cell count induced by both ECSOD and catalase, catalase or ECSOD relative to unprotected nerves. Asterisk (*) represents p<0.05 double asterisks (**) represents p<0.01, A represents astrocyte, M represents mononuclear inflammatory cell.

Mononuclear inflammatory cells and reactive astroglial cells predominantly involved the retrobulbar optic nerve of mice inoculated for EAE. ECSOD eyes inoculated with catalase had the greatest decrease in optic nerve cellularity. A representative light micrograph shows no inflammatory cells and a relatively normal complement of astroglial nuclei. (Figure 4A). In contrast, the unprotected nerves revealed many mononuclear inflammatory cells in addition to the astroglia (Figure 4B). The combined effects of in vivo scavenging of hydrogen peroxide by viral mediated catalase gene transfer and superoxide scavenging suppressed the optic nerve cell count by 27% with mean of 243 cells per 10^5^ μm^2^ (ECSOD OD) relative to 335 cells per 10^5^ μm^2^ (Wt OS) for untreated wild-type mice (p<0.01) (Figure 4C-D). This combined effect was greater than that of superoxide scavenging alone as ECSOD mice (ECSOD OS) had 17% less inflammation with a mean cell count of 276 cells per 10^5^ μm^2^ versus a mean of 335 cells per 10^5^ μm^2^ for wild-type mice (Wt OS; p<0.05). It was slightly greater than the solo effect of catalase mediated scavenging of hydrogen peroxide with a mean cell count of 258 cells per 10^5^ μm^2^ (Wt OD) relative to 335 cells per 10^5^ μm^2^ (Wt OS) that reduced inflammation by 23% (p<0.01).

### H_2_O_2_

**Figure 5:**
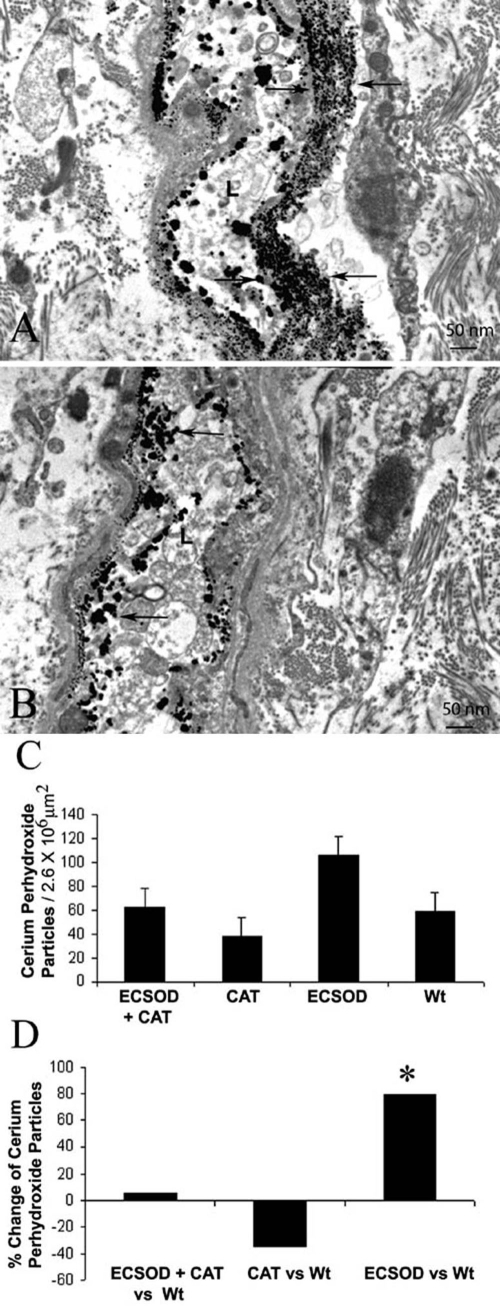
Reactive oxygen species in the optic nerve. Transmission electron micrographs show electron dense cerium perhydroxide (arrows) formed by the reaction of cerium chloride and endogenous hydrogen peroxide is more prominent in the optic nerves of animals protected by ECSOD (ECSOD OS; **A**) relative to unprotected nerves (Wt OS; **B**). The barplot shows mean cerium perhydroxide particle counts in the retrobulbar optic nerves protected by ECSOD and catalase (ECSOD OD), catalase (Wt OD), ECSOD (ECSOD OS) or unprotected EAE (Wt OS; **C**). Barplot (**D**) illustrates the relative changes in H_2_O_2_ reaction product counts in nerves treated with ECSOD and catalase, catalase or ECSOD relative to the unprotected nerves. Asterisk (*) represents p<0.05, L represents lumen.

Electron dense cerium derived H_2_O_2_ reaction product in the EAE optic nerve was greatest in the meninges of the optic nerve sheath, but was also evident in the optic nerve head and retrobulbar nerve, thus it was similar to the distribution of the ECSOD. Transmission electron micrographs show cerium perhydroxide reaction product in the lumen and perivascular space of ECSOD mice (Figure 5A) is increased relative to wild-type mice (Figure 5B). In the retrobulbar optic nerve of AAV-catalase gene inoculated eyes of ECSOD mice (ECSOD OD) a mean of 62 cerium perhydroxide particles per 2.6x10^6^ μm^2^ represented a 5% increase of H_2_O_2_ counts relative to 59 particles per 2.6x10^6^ μm^2^ for unprotected nerves of wild-type mice (Wt OD; Figure 5C-D). This slight difference was not statistically significant. Catalase gene inoculation into the eyes of wild-type mice (Wt OD) with a value of 38 particles per 2.6x10^6^ μm^2^ decreased H_2_O_2_ counts by 35% relative to the unprotected eyes of wild-type mice (Wt OS), but this difference was not statistically significant. However, cerium perhydroxide reaction product particles increased by 72% in the left eyes of ECSOD mice (ECSOD OS) with a mean of 106 particles per 2.6x10^6^ μm^2^ relative to unprotected left eyes of wild-type mice (Wt OS; p<0.01).

### Blood-brain barrier

**Figure 6:**
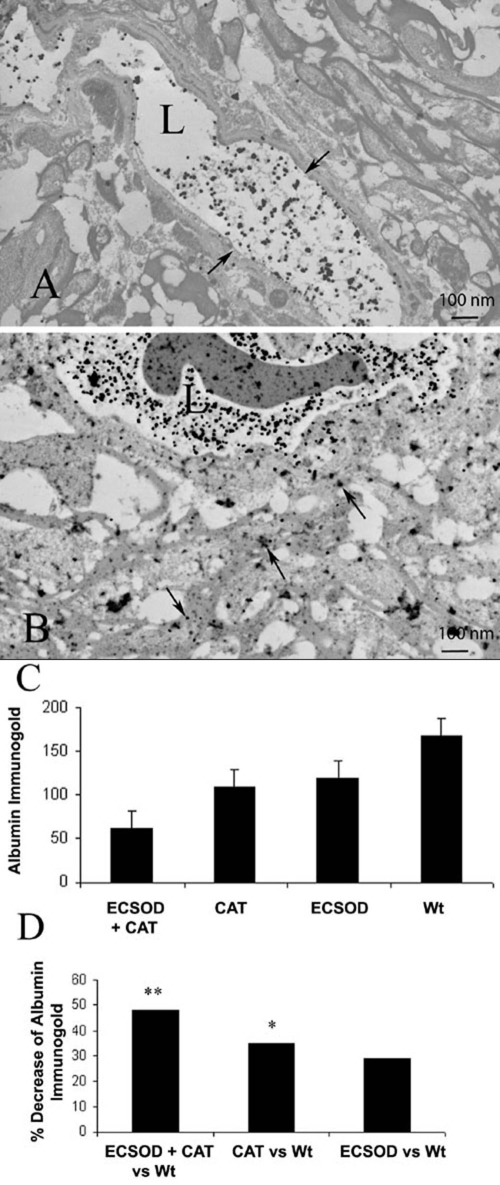
Restoration of blood-brain barrier integrity. Transmission electron micrographs show that the combined effects of catalase and ECSOD markedly decreased extravasation of serum albumin labeled by immunogold (arrows) from the vessel lumen into the perivascular space (**A**), relative to perivascular accumulation of labeled serum albumin in the unprotected optic nerve (**B**). The barplot shows mean extravasated albumin immunogold counts in the retrobulbar optic nerves protected by ECSOD and catalase (ECSOD OD), catalase (Wt OD), ECSOD (ECSOD OS) or unprotected EAE (Wt OS; **C**). Barplot (**D**) illustrates the decrease in extravasated albumin immunogold in nerves treated with ECSOD and catalase, catalase or ECSOD relative to the unprotected nerves. Asterisk (*) represents p<0.05, double asterisks (**) represents p<0.01, L represents lumen.

Disruption of the blood-brain barrier (BBB), a hallmark of optic neuritis and MS, was seen in all animals sensitized for EAE. A standard marker of BBB disruption is the extravasation of serum albumin that was detected by immunolabeling. Transmission electron microscopy of the optic nerves revealed albumin immunogold labeling in all animals with EAE. Extravasated albumin immunogold in the perivascular compartment located the foci of BBB disruption in EAE. Albumin immunogold confined to the intravascular compartment indicated normal integrity of the BBB. Figure 6 shows representative transmission electron micrographs of the optic nerve of ECSOD mice inoculated with rAAV-catalase exhibiting less extravasated serum albumin (Figure 6A) than the control left eyes of wild-type littermates in which accumulation of extravasated albumin immunogold in the perivascular space is evident (Figure 6B). AAV-delivered catalase to the right eyes of transgenic ECSOD mice (ECSOD OD) reduced disruption of the BBB by 63%, with a mean value of 62 extravasated immunogold particles per 2.6x10^6^ μm^2^ relative to the left eyes of wild-type mice (Wt OS) with a mean value of 167 extravasated immunogold particles per 2.6x10^6^ μm^2^ (p<0.01; Figure 6C-D). This combined effect was greater than a value of 108 extravasated immunogold particles per 2.6x10^6^ μm^2^ for catalase gene transfer to the eyes of wild-type mice (Wt OD) that suppressed BBB disruption by 35% relative to unprotected left eyes (Wt OS; p<0.05). It was also greater than 119 extravasated immunogold particles per 2.6x10^6^ μm^2^ for the solo effect of germ line increases in ECSOD (ECSOD OS) that suppressed disruption of the BBB by 29% relative to untreated left eyes of wild-type littermates (Wt OS; p<0.05). Thus, combined scavenging of superoxide by ECSOD and hydrogen peroxide by catalase gene transfer restored integrity to the BBB.

## Discussion

Our results demonstrate that most parameters of experimental optic neuritis were substantially ameliorated by genetically induced expansion of two key antioxidant enzymes, catalase and ECSOD. Previously, we had demonstrated the beneficial effects of detoxification of a single ROS (H_2_O_2_) by catalase gene monotherapy. However, catalase only suppressed demyelination by 38% [23,30]. Since multiple ROS are likely involved in the pathogenesis of EAE and MS, it was reasonable to expect that detoxifying several detrimental ROS would have the best suppressive effect on disease activity. Here by combining ROS scavenging with ECSOD and catalase, we achieved a 72% reduction in the most important parameter of EAE studied, myelin injury to the optic nerve. This result was substantially better than the solo effect of catalase or ECSOD. Dai and coworkers attempted this approach in an animal model of antigen-induced arthritis in rodents [40]. In their study combined scavenging with ECSOD and catalase did not have an additive protective effect on arthritis though each antioxidant gene had a protective effect as in our study, in which each also suppressed demyelination and disruption of the blood-brain barrier. Still, we found that the ECSOD did not exert a suppressive effect on optic nerve head swelling. Since optic nerve head edema is predominantly due to swelling of axons rather than accumulation of extracellular fluid, lack of intra-axonal ECSOD likely contributed to the relative lack of protection at the nerve head.

The linearity of the combined protective effect of ECSOD and catalase that suppressed blood-brain barrier disruption and demyelination in our study suggests that superoxide and hydrogen peroxide each has a direct detrimental effect on blood vessels and myelin in the EAE optic nerve. Still, superoxide and hydrogen peroxide can combine to generate a highly toxic metabolite, the hydroxyl radical. While scavenging of either reactant may then suppress hydroxyl radical formation, combined scavenging of either reactant may have no additional benefit. Clearly, this was not the case here. By detoxifying H_2_O_2_ to relatively nontoxic byproducts the solo effect of catalase was better than that of ECSOD that had the least protective effect. Several factors may have contributed to this result. Dismutation of superoxide by ECSOD increased extracellular H_2_O_2_ levels. Our prior publications have demonstrated the adverse impact of hydrogen peroxide on experimental optic neuritis [31,41]. Thus, accumulation of H_2_O_2_ in the extracellular compartment may have partially dampened the suppressive effect that dismutation of superoxide by ECSOD had on experimental optic neuritis. Still despite the increase in H_2_O_2_, ECSOD had a modest suppressive effect. In addition, the suppressive effect of ECSOD on experimental optic neuritis may have occurred perhaps by reducing generation of other pathogenic ROS. With less superoxide available to react with nitric oxide, levels of highly toxic peroxynitrite may have been suppressed and levels of nitric oxide increased [42]. Nitric oxide and peroxynitrite, formed by the reaction of nitric oxide and superoxide, also play a role in the pathogenesis of EAE and MS [43-45].

The antioxidant enzymes catalase and SOD can be used to target ROS for destruction, thus suppress tissue injury. However, there are limitations to the use of the proteins themselves as treatment agents in EAE and MS. First, the antioxidant enzyme (SOD) must be administered daily, even with conjugation of polyethylene glycol, to prolong the half-life of the enzyme [31,46]. Second, exogenous SOD or catalase are effective only during the periods of active BBB disruption when these high molecular weight proteins are able to penetrate the central nervous system (CNS) [47]. Finally, optic neuritis recurs in part due to the inability of the protein to cross the BBB after integrity is restored.^47^ Genetic augmentation of cellular defenses against superoxide and hydrogen peroxide helps surmount these limitations. Since transgene expression following delivery with the AAV vector is relatively long-lived, a single treatment may be sufficient. Still, treatment by intraocular injection incurs some risk, even with an AAV vector that is relatively nonpathogenic.

Our findings in acute EAE suggest that genetic amplification of cellular defenses against ROS may have a role in attenuating CNS injury associated with optic neuritis and perhaps MS. Determining whether comparable levels of protection are also maintained against the repeated demyelinating inflammation of chronic relapsing EAE may be key, if anti-ROS gene transfers are to be applied in a clinical setting. While the 72% suppressive effect on demyelination achieved here is promising, our next steps are to tackle the pitfalls inherent in dual AAV infection of the retina and optic nerve and to demonstrate a protective effect by AAV mediated transfer of both genes (catalase and SOD), or perhaps even a chimeric SOD [48] on chronic EAE.
